# Autophagy in Negative-Strand RNA Virus Infection

**DOI:** 10.3389/fmicb.2018.00206

**Published:** 2018-02-13

**Authors:** Yupeng Wang, Ke Jiang, Quan Zhang, Songshu Meng, Chan Ding

**Affiliations:** ^1^Department of Dermatology of First Affiliated Hospital, Dalian Medical University, Dalian, China; ^2^Cancer Center, Institute of Cancer Stem Cell, Dalian Medical University, Dalian, China; ^3^College of Veterinary Medicine, Yangzhou University, Yangzhou, China; ^4^Department of Avian Infectious Diseases, Shanghai Veterinary Research Institute, Chinese Academy of Agricultural Sciences, Shanghai, China

**Keywords:** negative-strand RNA virus, autophagy, virus replication, immune response, selective autophagy

## Abstract

Autophagy is a homoeostatic process by which cytoplasmic material is targeted for degradation by the cell. Viruses have learned to manipulate the autophagic pathway to ensure their own replication and survival. Although much progress has been achieved in dissecting the interplay between viruses and cellular autophagic machinery, it is not well understood how the cellular autophagic pathway is utilized by viruses and manipulated to their own advantage. In this review, we briefly introduce autophagy, viral xenophagy and the interaction among autophagy, virus and immune response, then focus on the interplay between NS-RNA viruses and autophagy during virus infection. We have selected some exemplary NS-RNA viruses and will describe how these NS-RNA viruses regulate autophagy and the role of autophagy in NS-RNA viral replication and in immune responses to virus infection. We also review recent advances in understanding how NS-RNA viral proteins perturb autophagy and how autophagy-related proteins contribute to NS-RNA virus replication, pathogenesis and antiviral immunity.

## Introduction to Autophagy

Macroautophagy (hereafter called autophagy) is an evolutionarily conserved process that includes the immersion and transport of cytosolic contents to the lysosome for degradation (Mizushima et al., 2002). According to the molecular explanations of autophagy and pertinent processes proposed recently by Galluzzi et al. (2017), there are two primary attributes that distinguish genuine, functional autophagic reactions, regardless of kind: (i) they include cytoplasmic material; and (ii) they peak with (and rigidly depend on) lysosomal degradation. The execution of autophagy involves more than 30 essential autophagy-related (Atg) genes (Mizushima and Klionsky, 2007). These Atg gene products participate in several distinct stages of autophagosome biogenesis: initiation with Atg1 (in yeast) or its equivalent Ulk1 (in mammals) complex, vesicle nucleation with the Atg6 (Beclin-1 in mammals)-class III phosphatidylinositol 3-kinase (PI3K) (hVps34) complex, vesicle elongation with the microtubule-associated protein light chain 3 (LC3) lipidation and vesicle fusion with SNX18 complex (Yin et al., 2016; Anding and Baehrecke, 2017; Davis et al., 2017; Kimmelman and White, 2017). Given that autophagy is a dynamic process, the rate at which lysosomes degrade autophagy substrates is a good indicator of such a global efficiency in autophagic responses, which is commonly known as “autophagic flux” (Loos et al., 2014). Galluzzi et al. (2017) suggested that autophagic flux refers to the rate at which the molecular machinery for autophagy identifies, segregates, and disposes of its substrates (through lysosomal degradation). Bafilomycin A1 (BafA1) is widely used to assess autophagy flux for its ability to prevent the fusion of autophagosomes and lysosomes. Although autophagy has robust cytoprotective functions in the majority of pathophysiological and experimental settings (Menzies et al., 2015), in some cases, excessive or uncontrolled levels of autophagy can trigger autophagy-dependent cell death, termed “autophagic cell death” (Liu and Levine, 2015).

Originally autophagy was identified as a response to starvation, and it was thought of as a non-selective digestion process; However, it is now evident that autophagy specifically degrade aggregated proteins and damaged cellular organelles via autophagy receptors that link cargo to growing autophagosomal membranes (Stolz et al., 2014). An autophagy receptor is defined by its ability to bridge cargo and autophagosomal membrane, leading to the engulfment of cargo by the autophagic membrane (Deng et al., 2017). The selective autophagy is generally mediated by autophagy receptors such as p62 /SQSTM1 for degradation of ubiquitylated protein aggregates (Pankiv et al., 2007; Ichimura et al., 2008), FAM134B for endoplasmic reticulum (ER) turnover (Khaminets et al., 2015; Chiramel et al., 2016), optineurin (OPTN) in xenophagy (Wild et al., 2011), NBR1 (neighbor of BRCA1) acting as an aggrephagy receptor (Kirkin et al., 2009). These autophagy receptors interacts with ATG8/LC3/GABARAP via the presence of a LC3 Interaction Region (LIR), also known as LC3 interaction motif (LIM) or Atg8 interaction motif (AIM), thus determining cargo recognition in selective autophagy (Birgisdottir et al., 2013; Wild et al., 2014). The autophagic destruction of invading pathogens, a process called xenophagy (Levine, 2005), involves autophagy receptors such as p62 and NDP52. Specifically, viral xenophagy (virophagy), as defined recently by Galluzzi et al. (2017) is anautophagic response targeting fully formed cytoplasmic virions or components. Mounting studies have demonstrated that viral infections may have complex interconnections with the autophagic process (Jackson, 2015; Lennemann and Coyne, 2015). Recent studies reported the three following main outcomes of these interactions: (i) autophagic machinery is utilized as a scaffold to promote virus replication; (ii) viruses disrupt or inhibit autophagic machinery to avoid restriction of their replication; and (iii) autophagy limit virus replication. Interestingly, replication of certain virus seems not to be affected either positively or negatively by autophagy, as observed in Drosophila C virus and human rhinovirus (Cherry et al., 2006; Brabec-Zaruba et al., 2007). Additionally, it is possible that certain virus may have different interactions with autophagy, dependent on the cell type. HIV exploits the autophagic machinery during early stages in replication, but in macrophages the viral nef protein blocks the conversion of autophagosomes to autolysosomes, thereby preventing the loss of virus to be proteolyticaly degraded (Kyei et al., 2009). Consequently, the modulation of autophagy by different viruses, virus strains or serotypes, may cause different effects on their host cells, thereby contributing to specific viral pathogenesis.

## Modulation of Autophagy by NS-RNA Viruses

Negative-strand (NS)-RNA viruses encompass some of the most significant human and animal pathogens extant, such as Ebola virus (EBOV), influenza virus (IAV), Nipah virus, Newcastle disease virus (NDV) and rabies virus (RABV). Similar to other viral pathogens, NS-RNA viruses overcome the host cell’s defense to ensure their own survival and propagation. Not surprisingly, autophagy plays a critical role in NS-RNA virus replication and/or infection. By dissecting the known interactions between NS-RNA viruses and the cellular autophagy machinery (**Table 1**), several distinct features are displayed as below:

**Table 1:** Summary of known interactions between *NS-RNA* viruses and autophagy.

Family/Virus	Interactions with autophagy	Impact of autophagy on virus replication and pathogenesis	Reference
**Arenaviridae** Lymphocytic choriomeningitis virus (LCMV)	Lack of Atg5 results in enhanced LCMV-specific CD8^+^ T cell responses *in vivo*. Macroautophagy machinery arranges T cell immunity by assisting the MHC class II but weakens the MHC class I-limited antigen presence	Autophagy is required for memory CD8^+^ T cell formation during an acute viral infection, autophagy in virus-specific CD8^+^ T cells is essential for cell survival and controlling a chronic viral infection.	Xu et al., 2014; Loi et al., 2016
**Bunyaviridae** Rift Valley fever virus (RVFV) Severe fever with thrombocytopenia syndrome virus (SFTSV) Sin Nombre Hantavirus (SNV)	LC3B expression is decreased during virus infection; induces autophagy via toll-7 /myD88 pathway. Induces autophagy Induces autophagosome formation in HUVEC.	Autophagy inhibited RVFV replication and infection. SFTSV NSs inhibited IFN responses independent of Atg7. Induction of autophagosome formation promoted virus replication; inhibition of the host autophagy machinery impedes viral replication. Autophagic clearance of Sin Nombre Hantavirus glycoprotein Gn encourages viral replication.	Popova et al., 2010; Moy et al., 2014; Santiago et al., 2014 Hussein et al., 2012
**Filoviridae** Ebola virus (EBOV) Marburg virus (MARV)	Involved in ER-phagy and/or chaperone-mediated autophagy Involved in chaperone-mediated autophagy	Autophagy is required for memory CD8^+^ T cell formation during an acute viral infection, autophagy in virus-specific CD8^+^ T cells is essential for cell survival and controlling a chronic viral infection. Chaperone-mediated autophagy activity of BAG3 portrays a particular host defense technique to thwart the capability of VP40 in encouraging efficient egress and spread of virus particles.	Chiramel et al., 2016 Liang et al., 2017
**Orthomyxoviridae** Influenza A virus (IAV) Influenza B virus (IBV) Infectious salmon anemia virus (ISAV)	Induces functional macroautophagy, but may blocks autophagosome degradation. H5N1 induces autophagic cell death in alveolar epithelial cells. Destruction of liver mitochondria in the influenza B virus model of Reye’s syndrome in mice Activates the cellular autophagy machinery.	Inhibition of autophagy decreases virus replication and ameliorates acute lung injury caused by H5N1 infection. Inhibition of autophagy reduced virus production.	Sun et al., 2012 Woodfin and Davis, 1986 Cottet et al., 2011
**Paramyxoviridae** Canine distemper virus (CDV) Human parainfluenza virus type 3 (HPIV3) Measles virus (MeV) Morbillivirus (MV) Newcastle disease virus (NDV) Peste des petits ruminants virus (PPRV) Respiratory syncytial virus (RSV) Sendai virus (SeV)	Induces autophagosome formation but prevent their subsequent fusion with lysosomes. Induced the accumulation of cytoplasmic autophagosomes by HPIV3 phosphoprotein (P)-mediated inhibition of autophagic flux MeV infection induces successive autophagy. Induces autophagy Induces autophagy *in vitro* and *in* *vivo*. Replication of PPRV is required for induction of autophagy. Induces autophagy in dentric cells; Sendai virus infection led to high levels of autophagy in wild-type MEFs; Inactivated Sendai virus induces autophagy in lung cancer cells via the PI3K/Akt/mTOR/p70S6K pathway	Autophagy by itself had a minimal effect on the preliminary replication efficiency, but it was needed for efficient spread. Inhibition of autophagy induction substantially lowered the spread of the virus. Inhibition of autophagic flux resulted in an increase in extracellular virion production. Matrix protein induces mitophagy that suppresses interferon responses. Sustained autophagy contributes to MeV infectivity. Morbilliviruses rapidly induce autophagy and require this induction for efficient cell-to-cell spread Autophagy enhances virus replication *in vitro* and *in* *vivo*. Induction or inhibition of autophagy enhances or decreases PPRV replication respectively. Autophagy-mediated dendritic cell activation is necessary for natural cytokine generation and APC function with respiratory syncytial virus reactions. Inhibition of RNase L-induced autophagy subdued viral growth in the first stages; in latter stages, autophagy encouraged viral replication.	Kabak et al., 2015 Ding et al., 2014; Ding et al., 2017 Richetta et al., 2013 Delpeut et al., 2012 Meng et al., 2012 Zhang et al., 2013 Morris et al., 2011; Reed et al., 2013, 2015 Siddiqui and Malathi, 2012; Zhang et al., 2015
Simian virus 5 (SV5)	Induces autophagy	SV5, which initiates pDC independent of replication, needs autophagy pathways.	Manuse et al., 2010
**Rhabdoviridae** Viral hemorrhagic septicemia virus (VHSV) Vesicular stomatitis virus (VSV) Rabies virus (RABV) Spring viraemia of carp virus (SVCV)	VHSV Gs induce an autophagic antiviral program in vertebrate cell lines. Both wild-type or UV-inactivated virus activates autophagy in DrosophilaS2 cells. VSV infection induces autophagy both in primary cells and in adult flies and in human cancer cells. Autophagy is immediately initiated via VSV infection, probably through the surface glycoprotein VSV G; therefore, initiation does not need viral replication. Induces autophagy Glycoprotein, rather than viral replication, activates the autophagy pathway	Regulating autophagy may be utilized to prevent and treat rhabdovira l infections. Autophagy plays an antiviral role in adult flies. At a low multiplicity of infection, the induction of autophagy throughout the first cycle of viral growth added to the suppression of virus replication. In future rounds of infection, autophagy encouraged viral replication. After VSV institutes infection, there is no further need for autophagy machinery throughout the latter stages of the infection cycle. Attenuated virus strain induces autophagosome accumulation in an *in vitro* model suggesting a role of autophagy in RABV pathogenesis SVCV utilized the autophagy pathway to facilitate its own genomic RNA replication	Garcia-Valtanen et al., 2014 Jounai et al., 2007; Lee J.G. et al., 2007; Shelly et al., 2009 Peng et al., 2016; Li et al., 2017 Liu et al., 2015

(i) Some NS-RNA viruses induce autophagy in the absence of viral replication. It is reported that Vesicular stomatitis virus (VSV), either wild-type or UV-inactivated, activated autophagy in Drosophila S2 cells (Wakana et al., 2013). Further study revealed that such activation of autophagy is most likely via the surface glycoprotein VSV G, and thus not requiring viral replication. In addition, inactivated Sendai virus (HVJ-E) induced autophagy in lung cancer cells via the PI3K/Akt/mTOR/p70S6K signaling pathway, and inducing autophagy enhanced HVJ-E-induced apoptosis (Zhang et al., 2015).
(ii) The autophagic induction pattern may differ between attenuated and virulent NS-RNA virus strains. In the case of measles virus (MeV), attenuated MeV strain induced a first transient wave of autophagy immediately upon infection via a CD46-Cyt-1/GOPC pathway (Joubert et al., 2009; Richetta et al., 2013). Interestingly, a second wave of autophagy was initiated after viral replication by the expression of the non-structural MeV protein C and was sustained overtime within infected cells (Richetta et al., 2013). Importantly, the sustained autophagy played a role in viral infectivity (Richetta et al., 2013). Mechanically, MeV protein C induced the next wave of autophagy throughout the interaction with immunity-associated GTPase family M (IRGM), a mediator of autophagy (Gregoire et al., 2011). Therefore, the reduced MeV strain prompts two waves of autophagy throughout infection in the specific molecular pathways. Of note, the harmful MeV strain was not able to prompt the initial CD46-dependent autophagic wave, but induced and exploited the later autophagic wave to replicate (Richetta et al., 2013). In addition, Xia et al. (2014) reported that MeV strain Edm utilized mitophagy to encourage viral replication by alleviating antiviral natural immune reactions. In the case of another NS-RNA virus, RABV, part of the family *Rhabdoviridae,* lessened SRV9 prompted more autophagosomes to gather rather than pathogenic CVS-11 in an *in vitro* model, suggesting a role of autophagy in RABV pathogenesis.

Notably, while the majority of the NS-RNA viruses induce autophagy for their own benefit during virus infection, some of them block autophagy especially at two checkpoints of the process, i.e., early during autophagosome formation and at the stage of autophagosome fusion with late endosomes or lysosomes. One example is the segmented RNA virus IAV which inhibits autophagosome maturation, leading to increased apoptotic cell death of infected cells (Gannage et al., 2009). The inhibition of autophagy flux by IAV is mediated by its matrix protein 2 (M2), which interacts with LC3 via its LIR motif. This interaction leads to the redistribution of LC3-coupled membranes to the cell membrane, which is esscential for IAV budding and transmission (Beale et al., 2014). Interestingly, canine distemper virus (CDV) and human parainfluenza virus type 3 (HPIV3) also block autophagosome fusion with lysosomes (Ding et al., 2014), which may promote different aspects of the viral replication cycle. Further studies revealed that HPIV3 blocks the SNAP29-STX17-mediated autophagosome-lysosome fusion via the HPIV3-P protein, which leads to improved viral release (Ding et al., 2014; Faure, 2014). However, the detailed mechanism needs to be further investigated.

Given that NS-RNA viruses target autophagy during infection, the underlying mechanism(s) by which NS-RNA viruses perturb autophagy remains elusive. A large body of evidence indicates that viral proteins play a critical role in NS-RNA virus-induced autophagy. For paramyxoviruses, viral glycoprotein-mediated membrane fusion triggers autophagy (Delpeut et al., 2012). In addition, in the case of Spring viraemia of carp virus (SVCV), part of the family *Rhabdoviridae,* SVCV glycoprotein, instead of viral replication, stimulates the autophagy pathway (Liu et al., 2015). Of importance, SVCV used the autophagy pathway to ease its own genomic RNA replication (Liu et al., 2015). A number of viral proteins encoded by separate NS-RNA viruses interact with cellular autophagy-related proteins, thereby contributing to the autophagy induction during virus infection. This will be discussed in detail in the following section.

## Role of Autophagy in NS-RNA Viral Replication

**FIGURE 1:**
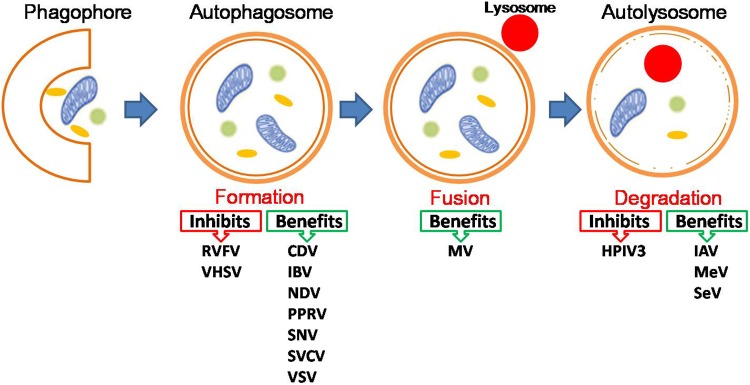
The pro-viral and anti-viral functions of autophagy during negative-stranded RNA viral infection. For detailed information, please see the main text. Abbreviations for viruses as below: Canine distemper virus (CDV), Human parainfluenza virus type 3 (HPIV3), Influenza A virus (IAV), Influenza B virus (IBV), Lymphocytic choriomeningitis virus (LCMV), Marburg virus (MARV), Measles virus (MeV), Morbillivirus (MV), Newcastle disease virus (NDV), Peste des petits ruminants virus (PPRV), Rift Valley fever virus (RVFV), Sendai virus (SeV), Sin Nombre Hantavirus (SNV), Spring viraemia of carp virus (SVCV), Vesicular stomatitis virus (VSV), Viral hemorrhagic septicemia virus (VHSV).

Given the known interplay between NS-RNA viruses and autophagy, how does autophagy impact on NS-RNA virus infection? Also, how do NS-RNA viruses benefit from autophagy? Accumulating evidence indicates that for most NS-RNA viruses, autophagy functions as a proviral mechanism in infected cells (**Figure 1** the pro-viral and anti-viral functions of autophagy during negative-stranded RNA viral infection), although this conclusion is largely based on *in vitro* investigations. Therefore *in vivo* studies on the role of autophagy in NS-RNA virus infection and pathogenesis, especially how autophagy regulates innate and adaptive immune responses to these pathogens, are of great interest and importance, particularly for those most significant human and animal pathogens, such as IAV and NDV. In the following sections, we discuss recent progresses in understanding the role of autophagy in NS-RNA viral replication cycle, ranging from virus entry up to egress.

IAV a member of the *Orthomyxoviridae*, causes severe morbidity and mortality in animals and humans. The complex interplay between IAV and host autophagy machinery has been extensively reviewed (Rossman and Lamb, 2009; Dumit and Dengjel, 2012; Yeganeh et al., 2013; Munz, 2014; Zhang et al., 2014; Tripathi et al., 2015). Generally, it is well recognized that IAV infection triggers autophagosome formation, but inhibits the fusion of autophagosomes with lysosomes. IAV proteins M2, hemagglutinin (HA) and non-structural protein 1 (NS1) are involved in initiating the formation of autophagosomes in infected cells (Gannage et al., 2009; Zhirnov and Klenk, 2013). A number of studies indicate that IAV strains such as H1N1, H3N2, H9N2 infection induce autophagy in HEK293 cells (Ren et al., 2015), murine macrophages (Law et al., 2010), monkey and canine kidney cells (Khare et al., 2013), human alveolar epithelial cells (Zhirnov and Klenk, 2013) and mouse dendritic cells (DCs) (Zang et al., 2016), which is reportedly involved in viral replication (Zhou et al., 2009; Lupfer et al., 2013). However, it should be noted that the role of autophagy in IAV replication is still debated in some settings. Law et al. (2014) reported that autophagy was not involved in H9N2/G1 virus replication in primary human blood macrophages, although autophagic responses played a role in IAV-induced CXCL10 and interferon-α expression in human macrophages. Nevertheless, the critical role of autophagy in IAV replication has been documented by a few *in vivo* studies (Gannage et al., 2009; Hahn et al., 2014). Using mouse models with recombination at the Atg5 locus in the distal respiratory epithelium (Hahn et al., 2014), Hahn et al. (2014) found that a 50% decrease in autophagy in the bronchoalveolar epithelium significantly attenuated influenza A/H3N2 viral replication, leading to improved lung structure and function and reduced morbidity and mortality following infection indicating that the reserve autophagic capacity in alveolar epithelia provides a replicative niche for IAV. However, these studies are not consistent with an early observation by Gannage’ et al. (2009) which showed that IAV replication progresses normally even in an autophagy-deficient cell line. Interestingly, another work in the mouse embryonic fibroblast (MEF) cell line has shown that IAV does not induce autophagy unless apoptosis is first inhibited (McLean et al., 2009), possibly explaining why Gannage’ et al. (2009) did not see any effect on viral replication when autophagy is inhibited in the MEF cell line. Supporting the research by Gannage’ et al. (2009) recently Sun et al. (2012) reported that the autophagy inhibition did not have a notable effect on the viral replication of IAV H5N1 *in vitro* or *in vivo*. *Thus,* the ultimate effect of autophagy on viral replication may be clarified only when examined in the context of the host immune system during an *in vivo* infection.

Interestingly, while it is generally believed that autophagic membranes might be utilized to provide the necessary means for viral envelope acquisition and/or to export virus particles from infected cells, it seems not in the case of IAV. Although IAV infection results in the accumulation of autophagic LC3-positive membranes (Gannage et al., 2009; Beale et al., 2014; Ren et al., 2015), which is delivered to the cell surface for IAV budding, LC3 is not incorporated into the budding virus particles.

Looking from the cellular autophagy side, it has been demonstrated that Atg genes have been implicated in viral entry, viral release, and cell death during IAV infection (Sun et al., 2012; Beale et al., 2014; Pirooz S. et al., 2014). Antonucci et al. (2015) reported that IAV protein synthesis was markedly reduced in mice pancreatic epithelial cells lacking the essential Atg7. Another study using Atg7^-/-^ MEFs by Liu et al. (2016) also found that autophagy deficiency significantly reduced the levels of IAV proteins, mRNA and genomic RNAs (vRNAs) without affecting viral entry. These studies together with data presented by Gannage et al. (2009) indicated that autophagy is utilized by IAV for accumulation of viral elements during IAV replication without affecting progeny virus production. It seems that autophagy is used by IAV to avoid the host’s defenses. *Thus,* the ultimate effect of autophagy on viral replication may be clarified only when examined in the context of the host immune system during an *in vivo* infection.

NDV a member of the *Paramyxoviridae*, is an important avian pathogen. Our study first reported that NDV FMW strain (NDV/FMW) induced autophagy in cell culture model, which plays a role in NDV replication (Meng et al., 2012). Subsequently we observed the conversion of LC3-I to LC3-II in heart, liver, spleen, lung, and kidney of NDV-infected chickens, confirmed the induction of autophagy *in vivo* (Sun et al., 2014). Of importance, modulation of the induction of autophagy with wortmannin, chloroquine, or starvation impacts NDV generation and pathogenesis in lung and intestine tissues in chickens (Sun et al., 2014), indicating that autophagy assists the replication of NDV in chicken cells and tissues and affects NDV pathogenesis (Sun et al., 2014). These studies are additionally supported by a current study that revealed that autophagosome formation was a requirement for NDV replication in chick embryo fibroblasts (Kang et al., 2017). In addition, recent work from our laboratory has further demonstrated that NDV nucleocapsid protein (NP) or phosphoprotein (P) was enough to prompt autophagy *in vitro*, involving activation of the ER stress-related unfolded protein response (UPR) pathways (Cheng et al., 2016). It should be noted that although the above studies indicate that NDV-induced autophagy benefits NDV replication, the detailed mechanism remains elusive.

Given that some NDV strains such as FMW, which is used in our *in vitro* study, are onlytic, the role of autophagy in oncolytic NDV replication is investigated by several studies. We found that NDV/FMW triggered autophagy in paclitaxel-resistant A549 lung cancer cells via dampening the class I PI3K/Akt/mTOR/p70S6K pathway whereas it attenuated the autophagic process in cisplatin-resistant A549 cells through the activation of the negative regulatory pathway (Jiang et al., 2014). Surprisingly, autophagy modulation does not increase virus progeny in these drug resistant cells, which is in consistent to the observation from NDV-infected parent cells (Jiang et al., 2014). In addition, Wei group found that NDV La Sota strain induced autophagy and preserved autophagic flux in non-small cell lung cancer cells and mitophagy promoted NDV replication by blocking intrinsic apoptosis (Meng et al., 2014). Interestingly, our recent investigation showed that NDV/FMW promotes autophagy flux in lung cancer cell spheroids (Hu et al., 2015). The difference in the autophagy pattern induced by oncolytic NDV may be ascribed to the different virulence between these NDV strains.

VSV a member of the *Rhabdoviridae*, is a prototypic non-segmented negative-strand RNA virus. In the case of VSV, the role of autophagy in virus replication seems to be controversial. An early study by Jounai et al. (2007) showed that either Atg5- or Atg7-deficient MEFs, in which Atg5-Atg12 conjugation is impaired, were resistant to VSV replication, suggesting that autophagy favors VSV replication. Further analysis indicated that the Atg5-Atg12 conjugate immediately interacts with the retinoic acid-inducible gene I (RIG-I) and IFN-β promoter stimulator 1 (IPS-1); this molecular connection causes the inhibition of type I IFN production and allows VSV replication inside the infected cells. On the contrary, Shelly et al. (2009) observed that VSV infection prompts autophagy in the primary cells and in adult flies, and suppression of autophagy causes greater viral replication and pathogenesis in cells and animals, supporting a an essential anti-viral role of autophagy in Drosophila against VSV (Shelly et al., 2009). The underlying mechanism was investigated in a subsequent study by the same group, who reported that Toll-7 interacted with VSV at the plasma membrane and induced antiviral autophagy in a drosophila model (Nakamoto et al., 2012). Interestingly, early to the investigation by Shelly et al. (2009) and Lee H.K. et al. (2007) observed that pharmacological inhibitors of autophagy, 3-methyladenine (3-MA) and Wortmannin inhibited VSV recognition in plasmacytoid dendritic cells (pDCs) while they specifically inhibited autophagosome formation without affecting viral entry and infection (Lee H.K. et al., 2007). Considering these studies are performed in different animal models, they seemingly indicate that the role of autophagy in VSV replication may be spices dependent. Similar to NDV, VSV is known as an oncolytic virus. The role of autophagy in VSV infection and oncolysis was investigated by several labs. Schache et al. (2009) reported that oncolytic VSV induced autophagy in a variety of cancer cells, and autophagy seems not play a role in VSV virotherapy while the role of autophagy in virus replication has not been investigated in these settings. However, a recent study by Shulak et al. (2014) showed that either 3-MA treatment or genetic ablation of the autophagic marker Atg5 decreased VSV replication and oncolysis in human prostate cancer PC3 cell, consisting with the study by Jounai et al. (2007) in mouse model. Taking together, the precise mechanism underlying the conflicting role of autophagy in VSV infection remains to be clarified, although VSV G protein-mediated autophagy during virus infection has been investigated in a number of studies, which we will discuss below.

While autophagy is shown to promote virus replication for most NS-RNA viruses, for some NS-RNA viruses like CDVand MeV, autophagy may just favor virus spread rather than replication (Richetta et al., 2013; Kabak et al., 2015). CDV and MeV prompt autophagy at the initial infection stages. For attenuated/vaccinal MeV but not virulent MeV, the early wave of autophagy induction upon virus infection may contribute to virus entry (Richetta et al., 2013). However, the late wave of autophagy induced by the virulent but not the non-replicative UV-treated MeV plays a critical role in virus replication (Richetta et al., 2013; Guirimand et al., 2015). Moreover, a very late autophagy induction by the cell–cell fusion also contributes to MeV replication (Herschke et al., 2007). Interestingly, although peste des petits ruminants virus (PPRV), along with CDV and MeV, is classified in the genus Morbillivirus within the family Paramyxoviridae, PPRV uses the autophagy machinery to ease its replication in host cells (Zhang et al., 2013), indicating that the role of autophagy in virus replication and/or spread is virus dependent.

Notably, other patterns of selective autophagy such as ER-phagy also play roles in NS-RNA viral replication. FAM134B is the selective autophagy receptor for endoplasmic reticulum turnover (Khaminets et al., 2015; Mochida et al., 2015). Using FAM134B^-/-^ MEFs, Chiramel et al. (2016) recently demonstrated that ER-phagy limits EBOV replication in mouse cells. Similarly, SQSTM1/p62-mediated mitophagy enhances MeV replication by mitigating DDX58/RIG-I-like receptor signaling (Xia et al., 2014). Altogether, these studies highlights the role of selective autophay in NS-RNA viral replication.

The role of autophagy in NS-RNA viral replication is generally mediated by autophagy-related proteins. Accumulating evidence reveals that autophagy-related proteins are targeted by NS-RNA viruses during infection and importantly these proteins play a role in NS-RNA virus infection and pathogenesis. Using yeast two-hybrid and bioinformatic analysis, Gregoire et al. (2012) determined the molecular interactions between 44 autophagy-associated proteins and 83 viral proteins belonging to five different RNA virus families including two families of NS-RNA viruses, *Paramyxoviridae* and *Orthomyxoviridae* (Gregoire et al., 2012). This interactome revealed that IRGM is the most targeted autophagy-associated protein (Gregoire et al., 2012). Downregulation of IRGM expression prevented autophagy induction by MeV, and impaired viral particle production. Interestingly, the expression of IRGM-interacting MeV-C protein was sufficient to induce autophagy through an IRGM dependent pathway, which could contribute to the facilitation of the syncytia formation. Together, the study by Gregoire et al. (2012) demonstrated that IRGM is a common target of RNA viruses that subvert the autophagy network. In addition, the human inhibitory complement receptor CD46, a type I glycoprotein expressed by all nucleated human cells, has been reported to be a direct inducer of autophagy and binds multiple pathogens, including MeV (Persson et al., 2010). It should be pointed out that the effect of certain autophagy-related proteins on NS-RNA viruses may be independent of autophagy. For instance, UV-radiation resistance-associated gene (UVRAG), an autophagic tumor suppressor, is required for the entry of the prototypic negative-strand RNA virus, including IAV and VSV, by a mechanism independent of IFN and autophagy (Pirooz S.D. et al., 2014).

In addition, autophagy-related proteins may directly interact with NS-RNA viral proteins to affect virus spread and/or replication. Among them, Beclin-1 is often targeted to induce autophagy by viral proteins such as IAV M2 protein. BCL2 associated athanogene 3 (BAG3), a regulator chaperone-mediated autophagy, sequestered EBOV and Marburg (MARV) viruses VP40 away from the site of budding at the plasma membrane, thereby counteracting the function of VP40 in promoting efficient egress and spread of virus particles (Liang et al., 2017). In addition, diverse autophagy receptors contribute to NS-RNA virus replication and/or pathogenesis. Chiramel et al. (2016) found that FAM134B, the selective autophagy receptor for endoplasmic reticulum turnover, inhibits replication of Ebola virus strains in MEFs via targeting the production of viral proteins GP and VP40 and nucleocaspid lattices. Interestingly, in the case of MeV, the autophagy receptors NDP52 and T6BP, but not OPTN, impacted MeV replication, although independently, and possibly through physical interaction with MeV proteins including MeV-N, MeV-C or MeV-V (Petkova et al., 2017).

## NS-RNA Virus Proteins Target Autophagy Machinery

Growing evidence indicates that both viral proteins and host cellular autophagy-related proteins play critical roles in the interplay between NS-RNA virus and autophagy, thereby contributing to virus replication and/or pathogenesis. In one hand, viral proteins such as IAV proteins including M2 have been shown to interact with autophagy-related proteins to affect the life of virus in infected cells. IAV proteins M2, HA, and NS1 take part in starting the formation of autophagosomes in infected cells (Gannage et al., 2009; Zhirnov and Klenk, 2013). Using yeast two-hybrid technique Gregoire et al. (2011) examined the interactions between 9 IAV proteins and 44 human autophagy-associated proteins and shown the associations of NP protein with Atg4C, BNIP3, and GOPC proteins, NS1with Atg5 and GOPC, NS2 with Atg5, Atg9A, IRGM and UVRAG, PB1-F2 with Atg5 and IRGM, PB2 with SQSTM1, and M2 with Beclin-1. However, given these interactions among IAV proteins and autophagy-related proteins, besides M2, whether other IAV proteins regulate autophagy during IAV infection is largely unknown. Recent study by Zhirnov and Klenk (2013) showed that NS1 expressed alone was unable to upregulate autophagy, whereas HA and M2 were. However, NS1 stimulated autophagy indirectly by up-regulating the synthesis of HA and M2, thus NS1 and HA along with M2 are involved in stimulation of autophagy in infected cells (Zhirnov and Klenk, 2013). Nevertheless, the precise roles of these IAV proteins in the interaction between IAV and autophagy need to be further confirmed in *in vivo* studies. Below we discussed how three important viral proteins from IAV, MeV and VSV play critical roles in virus replication and/or pathogenesis via the interaction with the cellular autophagic machinery.

Matrix 2 M2 functions as a proton-selective ion channel and regulates IAV assembly and budding. Gannage’ et al. (2009) first reported that the ability of IAV to evade autophagy depends on the M2 ion-channel protein, expression of M2 was enough to reproduce the phenotype of autophagosomes gathering and obstructing autophagosome maturation. The functional result of obstructing autophagosome development by M2 is a larger susceptibility of IAV-infected cells to apoptosis (Gannage et al., 2009). Further analysis demonstrated that the first 60 amino acids of M2 enable associated with Beclin-1 and were sufficient for inhibition of autophagy (Gannage et al., 2009). Interestingly, the role of proton channel activity of M2 in IAV-perturbed autophagy is debated, as Gannage’ et al. (2009) reported that proton channel activity of M2 was not involved in IAV-induced autophagy arrest whereas Ren recently showed that M2 proton channel activity was involved in blocking the fusion of autophagosomes with lysosomes (Gannage et al., 2009; Ren et al., 2015). In addition to the above-mentioned mechanism, another possible molecular mechanism underlying how M2 regulates host cellular autophagy machinery was revealed by Beale et al. (2014). They found that the cytoplasmic tail of IAV M2 interacts directly with the autophagy protein LC3 and promotes LC3 relocalization to the plasma membrane (Beale et al., 2014). Importantly, mutations in M2 that abolish LC3 binding reduced filamentous virion budding and stability *in vitro* (Beale et al., 2014), although how this process enhances virion stability remains to be explored. Therefore, IAV infection may subvert autophagy to boost transmission to new cells and/or hosts by increasing virion stability.

The well-studied interaction between M2 and LC3 highlights the importance of the role of LIR motif in viral proteins in virus-perturbed autophhagy. In deed, a very recent study revealed that a large number of potential LIR sequences contained within the viral proteins from over 16000 viral sequences and 2500 viral species (Jacomin et al., 2017). However, whether these LIRs are bona fide and functional sequences that are important for the viral life cycle remains to be investigated although some of them have been identified as functional player in viral life.

VSV surface glycoprotein G VSV-G is involved in receptor recognition at the host cell surface and triggers membrane fusion after endocytosis of the viron. Shelly et al. found that UV-inactivated VSV or VSV-G virus-like particles induced autophagy in DrosophilaS2 cells, indicating that VSV-G alone is adequate to induce autophagy without any additional virus components or viral genome replication (Shelly et al., 2009). But how VSV-G is recognized? The authors suggest that either a *Drosophila* TLR or a viral receptor may be the trigger (Shelly et al., 2009). This was confirmed in a subsequent study which indicated that VSV was recognized by Toll-7, which interacted with VSV virions at the plasma membrane, and this recognition was required for the induction of antiviral autophagy, suggesting that VSV-G may serve as the pathogen-associated molecular pattern (PAMP) to be recognized by Drosophila TLR (Shelly et al., 2009). However, it cannot rule out that VSV-G or VSV may interact with other cell surface receptors in mammalian cells to induce autophagy.

C protein MeV protein C is a non-structural protein not present within the virion and suppresses the host innate immune response by interfering with IFN signaling pathways. Throughout MeV infection, the MeV-C protein induced autophagy in infected cells via IRGM-dependent pathway, which could add to easing of syncytia formation (Gregoire et al., 2011). Importantly, MeV-C expression is a necessary requirement for the effective prompting of another MeV-induced autophagy wave (Richetta et al., 2013). Although MeV was lacking C protein expression, which does not prompt autophagy in infected mononucleated cells, autophagy was still noted in syncytia, suggesting that the expression of MeV-C protein is not critical to prompt autophagy syncytia. Thus, other proteins of MeV may be involved in this process. Indeed, it has recently been suggested that MeV could prompt autophagy via a fusogenic-dependent mechanism that necessitates the coexpression of MeV-F and MeV-H proteins (Delpeut et al., 2012).

Together, although a few viral proteins encoded by NS-RNA viruses have been revealed their roles in regulating autophagy *in vitro,* further investigation is needed to determine whether these proteins modulate autophagy *in vivo* infection models in the context of their complete genomes, and importantly, to what extent, viral protein-modulated autophagy contributes to virus replication and/or pathogenesis.

## Role of Autophagy in Immune Reponses to NS-RNA Virus Infection and in Pathogenesis

Given that selective autophagy target intracellular pathogens for destruction, it is now regarded as a critical aspect of the innate immune response. In fact, both innate as well as adaptive immune responses to virus infections are modulated by autophagy (Paludan et al., 2005; Schmid and Munz, 2007; English et al., 2009; Kuballa et al., 2012; Richetta and Faure, 2013; Paul and Munz, 2016). Below by dissecting the role of autophagy in IAV pathogenesis, we describe the role of autophagy in immune responses to IAV.

The role of autophagy in IAV pathogenesis has been examined *in vivo*. Study by Sun et al. (2012) demonstrated that autophagic cell death was responsible for the acute lung injury and the high mortality rate (60%) induced by IAV H5N1 in a mouse model. Importantly, H5N1, but not seasonal H1N1, induced autophagic cell death in alveolar epithelial cells (Sun et al., 2012), suggesting that the interplay between IAV and host autophagy machinery may be virus strain and virus virulence- dependent, since the seasonal H1N1 is a low pathogenic strain. A recent *in* *vivo* study by Lu et al. (2016) demonstrated that mice deficient of an Atg gene Epg5 exhibited elevated baseline innate immune cellular and cytokine-based lung inflammation and were resistant to lethal IAV infection. Similar results were observed in myeloid cells deletion of autophagy genes including Atg14, FIP200, Atg5, and Atg7 (Lu et al., 2016). These studies suggest that autophagy can mediate the anti-IAV immunity, thereby contributing to IAV-induced pathogenesis. The underlying mechanism is investigated by several studies. Early studies found that autophagy could ease the effective antigen cross-priming of IAV-specific CD8^+^ T cells (Uhl et al., 2009), and participated in the generation of CXCL10 and IFN-α via macrophages upon H1N1 virus infection (Law et al., 2010). The latest study found that mice lacking Atg5 or Atg7 have defective effector responses to IAV infection (Puleston et al., 2014). Recently Schlie et al. (2015) showed that in reaction to influenza infection, Atg5^-/-^ CD8^+^ T cells had a lowered ability to achieve the peak effector reaction and they were not able to sustain cell viability throughout the effector phase, indicating that effector CD8^+^ T cells need autophagy to subdue apoptosis and retain survival in reaction to a viral infection (Schlie et al., 2015).

In addition to macrophages and T cells, other immune cells such as DCs also contribute to the effect of autophagy in antiviral immunity upon IAV infection. Chen et al. (2014) recently found that mice with B cell-specific removal of Atg7 (B/Atg7^-/-^ mice) demonstrated typical primary antibody reactions following immunization against influenza, but they did not produce protective secondary antibody reactions when challenged with IAV, causing high viral loads, broad lung damage, and raised the fatality rate (Chen et al., 2014), indicating that autophagy has a pivotal part in retaining the memory B cells that protect against influenza virus infection. In addition, Zang recently reported that H1N1 virus infection-induced autophagy in DCs has an intensive part in DC-regulated immune reactions (Zang et al., 2016). They found that autophagosomes delivered H1N1 viruses into lysosomes to initiate the TLR signaling pathway and that autophagic deficiency damaged the antigen-presenting capability of DCs and their ability to prompt Th cell differentiation and inhibit the MHC-I cross-presentation of DCs upon H1N1 infection (Zang et al., 2016). In addition, autophagy-deficient BMDCs were weakened in their capability to prompt H1N1-specific natural and adoptive immune reactions *in vivo* (Zang et al., 2016).

The induction of the cytokine storm has been believed to be a primary cause of death in IAV H1N1-infected patients (Law et al., 2010). Of note, autophagy has been revealed to be critical for the generation of cytokines from innate immune cells. Using H5N1 pseudotyped viral particles (H5N1pps), Pan H.Y. et al., 2014 showed that obstructing autophagy with 3-MA (an autophagy inhibitor) or siRNA knockdown of autophagy-related genes (Beclin1 and Atg5) greatly lowered H5N1pps-induced proinflammatory cytokines and chemokines, including IL-1β, TNF-α, IL-6, CCL2, and CCL5, both *in vitro* and *in vivo* (Pan H. et al., 2014), suggesting the crucial part of autophagy in H5N1pps-triggered inflammatory responses. In line with the above observations, Lu et al. (2016) reported that Epg5 deficiency in mice caused an increase in IL-1β and IL-13 protein in lung macrophages, conferring influenza resistance. Interestingly, IAV could be sensed by NOD2, a member of the NLR family. The NOD2-RIPK2 axis could phosphorylates ULK1, leading to enhanced mitophagy which prevents excessive inflammasome activation (Lupfer et al., 2013). Therefore, these studies suggest that autophagy regulates inflammasome activity, thereby providing new possible targets for immunotherapy to combat IAV infection.

In addition to playing a role in IAV immunity, autophagy-related proteins, such as Atg5 and Atg7, also play a role in the generation of both innate and adaptive immune responses to other NS-RNA viruses (Subauste, 2009). Lee H.K. et al. (2007) demonstrated that in pDCs, Atg5 was required for the transport of VSV and Sendai virus (SV) genetic material starting at the cytoplasm and went into the endosomal compartment in which the ssRNA sensor TLR7 remains (Lee H.K. et al., 2007). This resulted in the production of IFN-αinnate response to the viruses (Lee H.K. et al., 2007), indicating that the generation of type I interferons via pDCs is an important part of the natural immunity to the virus that can be moderated by the autophagy protein Atg5. However, it was documented that autophagy proteins, including Atg5, can negatively moderate type I interferon generation, thereby promoting viral replication (Jounai et al., 2007). Atg5-deficient MEFs were resistant to VSV replication because of raised Type I IFN generation in reaction to VSV genetic material (Jounai et al., 2007). Mechanically, after infecting with VSV, the Atg5-Atg12 conjugate hinders type I interferon generation by attaching toRIG-I and IPS-1 via the caspase recruitment domains (Jounai et al., 2007). Subsequently another study indicated that Atg5 deletion in MEFs increased Type I IFN production, thus conferring resistance to VSV infection (Tal et al., 2009). As for Atg7, Chen et al. (2014) recently showed that when challenging with IAV in mice with B cell-specific deletion of Atg7 (B/Atg7^-/-^ mice) with IAV, these mice failed to generate protective secondary antibody responses although normal primary antibody responses after immunization against IAV was induced, suggesting that autophagy is necessary for the survival of virus-specific memory B cells in mice and the maintenance of protective antibody responses required to combat IAV infection (Chen et al., 2014). Therefore, the role of autophagy-related proteins in the antiviral immunity needs to be further investigated, since increasing evidence indicates that autophagy proteins regulate cellular responses other than autophagy.

Of note, autophagy-related proteins also target the innate immunity components, such as RIG-1, to add to the replication of particular NS-RNA viruses (Lei et al., 2012; Rey-Jurado et al., 2015). Jounai et al. (2007) reported that the overexpression of a mutant of RIG-1 prompted the initiation of NF-κB and IFN-β, which was subdued via the Atg5-Atg12 complex. Interestingly, this suppression appeared to be reliant on the infection with VSV and was documented to prompt a conformational alteration in RIG-1 (Jounai et al., 2007), suggesting that Atgs activated by VSV could modulate RIG-1.

Given the role of autophagy-related proteins in antiviral adaptive immunity during NS-RNA virus infection, autophagy could be exploited by NS-RNA viruses to evade host immunity (Rey-Jurado et al., 2015). Using a Beclin-1^+/-^ mouse model, Reed et al. (2013) revealed that the depletion of autophagy function resulted in lowered MHC-II expression following RSV infection and the inability to generate IFN-γ and IL-17, and as a result, hindering DC maturation and the start of an efficient antiviral adaptive immune response against this virus. In addition, the expression of granzyme B in CD8^+^ T cells has been demonstrated to be downregulated in the Beclin-1^+/-^ mice (Reed et al., 2013).

## Concluding Remarks

The increasing evidence on the role of autophagy in NS-RNA virus replication and pathogenesis suggests that modulation of autophagy may represent a novel therapeutic strategy against virus infection, such as IAV. However, several important issues remained to be explored:

(i) Most of the data regarding the interplay between NS-RNA viruses and host autophagy machinery are obtained in cell cultural model; the question remained whether such interactions are present or relevant during infections *in vivo*. (ii) How different NS-RNA viruses perturb autophagy to augment replication and pathogenesis? Reversely, how autophagy regulates the various aspects of NS-RNA virus replication and propagation? Obviously, our current understanding of the role of the autophagy machinery in the propagation and control of virus infections, the ability of viruses to co-opt the cellular autophagic pathway to establish virulence *in vivo* is in its infancy. For example, for NS-RNA viruses, the role of autophagy in the assembly of viral components and budding of viral particles remains largely unknown. (iii) It is not known whether the *in vitro* or animal trials of autophagy modulators can be translated into useful therapeutics against viral infections in humans. Particularly, such approaches should not only enhance augment direct antiviral activity against NS-RNA virus infection, but could also augment natural acquired immune responses and vaccination strategies. Taking these points into consideration, targeting autophagy may lead to the development of a new class of specific antiviral therapies for the treatment of NS-RNA viruses-related diseases.

## Author Contributions

SM designed the study. YW, KJ, and SM wrote the manuscript and reviewed the manuscript. KJ and QZ edited the manuscript. CD participated in developing the hypothesis. All authors listed approved the version to be published and have made a substantial and intellectual contribution to the work.

## Conflict of Interest Statement

The authors declare that the research was conducted in the absence of any commercial or financial relationships that could be construed as a potential conflict of interest.
